# First report of *mecC* MRSA in human samples from Austria: molecular characteristics and clinical data

**DOI:** 10.1016/j.nmni.2014.11.001

**Published:** 2014-11-12

**Authors:** H. Kerschner, E.M. Harrison, R. Hartl, M.A. Holmes, P. Apfalter

**Affiliations:** 1) National Reference Center for Antimicrobial Resistance and Nosocomial Infections, Institute for Hygiene, Microbiology and Tropical Medicine, Elisabethinen Hospital, Linz, Austria; 2) Department of Veterinary Medicine, University of Cambridge, Cambridge, United Kingdom

**Keywords:** Austria, cefoxitin, Genspeed, *mecC*, MLST, MRSA, PCR, *Staphylococcus aureus*, whole genome sequencing

## Abstract

Reports of *mecC* methicillin-resistant *Staphylococcus aureus* (MRSA) strains have been published from several European countries. We describe the first six *mecC* MRSA isolates of human origin from Austria and report the application of a rapid PCR test. Candidate isolates (*n* = 295) received between 2009 and 2013 were investigated phenotypically by cefoxitin screening and streaking on ChromID MRSA plates. The presence of *mecC* was confirmed in six isolates from blood cultures, wound swabs and screening samples of four female and two male patients (age range 7–89 years) by an in-house PCR method and the new Genspeed MRSA test (Greiner Bio-One, Kremsmünster, Austria). The *mecC* MRSA were further characterized by whole genome sequencing, multilocus sequence and *spa* typing. Antimicrobial susceptibility testing was performed by Eucast disk-diffusion method and Vitek 2. The six *mecC* MRSA isolates were from two clonal lineages (CC130, including a new single-locus variant, and CC599) and four different *spa* types (t843, t1535, t3256, t5930). Analysis for virulence factor genes yielded *lukED, eta, etd2* and *edin-B* (CC130 isolates) and *tst, lukED, eta* and *sel* (ST599 isolates). The Genspeed MRSA test identified *mecC* in all isolates whereas Vitek 2 failed to detect methicillin resistance in one isolate. The strains were susceptible to a wide range of non-β-lactam antibiotics. All patients were successfully treated or decolonized. *mecC* MRSA are present in Austria as colonizers but may also cause infections. Thus, laboratories must choose appropriate test methods such as cefoxitin screening and confirmation using molecular assays specifically targeting *mecC*.

## Introduction

Methicillin-resistant *Staphylococcus aureus* (MRSA) isolates carrying the *mecA* homologue *mecC* have been reported from all over Europe [1–8]. They may be detected phenotypically by routine cefoxitin screening and by PCR using specific primers; however, standard molecular diagnostic systems based on amplification of *mecA* fail to recognize these strains due to nucleic acid divergences between *mecA* and *mecC*. Published clinical data concerning *mecC* MRSA in humans include reports about colonization as well as skin and soft tissue infections [4], but also include fatal bacteremia [7] and osteomyelitis [9]. Thus, reliable detection of these strains in diagnostic microbiology routine is important [10].

The National Reference Centre for Antimicrobial Resistance and Nosocomial Infections at the Elisabethinen Hospital Linz receives bacterial isolates of human origin for identification, confirmation and typing from Austrian laboratories. Its strain collection contains over 5000 isolates of *Staphylococcus* spp. many of which have been extensively studied and typed using molecular methods [11–14]. We searched this strain collection for *S. aureus* carrying *mecC* using the conventional phenotypic approach followed by molecular confirmation with an in-house PCR method as well as one of the first commercially available systems also able to detect *mecC*, the Genspeed MRSA test (Greiner Bio-One, Kremsmünster, Austria). In addition, clinical and molecular typing data on four *mecC*-positive isolates detected as part of routine screening are presented, describing for the first time the presence of *mecC* MRSA in human samples from Austria.

## Materials and Methods

### Bacterial isolates, phenotypic and molecular antibiotic susceptibility testing and typing

Candidate *S. aureus* isolates (*n* = 295) that had tested negative for *mecA* and positive for *femA* using previously published primer sets between the years 2003 and 2012 were chosen from the strain collection [15,16]. Additionally, four strains received from Austrian laboratories in 2012–2013 for further testing regarding *mecC* were included in this study. Strains were subcultured overnight on trypticase soy agar containing 5% sheep’s blood (Oxoid, Wesel, Germany) at 36 ± 1°C in an aerobic atmosphere. Species identification of all isolates was done by matrix-assisted laser desorption-ionization time-of-flight (MALDI-TOF) mass spectrometry using the IVD MALDI Biotyper (Bruker Daltonik, Bremen, Germany) and the Vitek 2 system (bioMérieux, Marcy l’Etoile, France).

Susceptibility testing was performed according to the Eucast disk-diffusion method. All strains were screened phenotypically for methicillin susceptibility using cefoxitin 30 μg disks in quintuplicate. They were also inoculated onto ChromID MRSA agar plates (bioMérieux) that were read after 24 hours of incubation at 37°C. The broader antimicrobial susceptibility of *mecC* MRSA for a panel of substances (Table 1) was assessed by disk diffusion testing, and for selected substances, minimum inhibitory concentrations (MICs) were determined by gradient diffusion testing (Etest; bioMérieux) (Table 1). The susceptibility profiles of the *mecC* MRSA were also assessed using Vitek 2 Gram-positive antimicrobial susceptibility testing cards (bioMérieux).

All isolates showing a mean cefoxitin zone diameter <22 mm and/or growth on selective media underwent confirmatory PCR testing using a protocol published by Stegger *et al.* [17] to detect *mecA*, *mecC* and *lukF-PV* after extraction of bacterial DNA with InstaGene Matrix (BioRad, Hercules, CA, USA), with modified primer concentrations. Additionally, a commercially available rapid PCR system, the Genspeed MRSA (Greiner Bio-One, Kremsmünster, Austria) was evaluated. This system uses rapid bacterial lysis followed by conventional PCR and detection of *mecA* and *mecC*, including specific probes for *S. aureus*, via hybridization on a chip.

Isolates carrying *mecC* were further analyzed by pulsed-field gel electrophoresis of *Sma*I restriction fragments. They were also typed by amplification of the polymorphic X region of the protein A gene (*spa*) followed by sequencing and assignation of the *spa* type [18]. Whole genome sequencing of *mecC*-positive isolates was performed as previously described [19] to confirm their *mecC* gene status, to determine their multilocus sequence type (ST) and to identify virulence factor and antibiotic resistance genes. Nucleotide sequences have been deposited in the European short-read archive as ERR387183, ERR387184, ERR387185, ERR387090, ERR 490434 and ERR490433 for isolates 4402, 5127, 5590, 5625, 5675 and 5752, respectively.

### Clinical information

Clinical information on patients with *mecC* MRSA was obtained from medical records retrospectively. Consent of the local ethics committee was provided (C-60-13).

## Results

All strains were confirmed as *Staphylococcus aureus* with MALDI-TOF scores of >2.0. Phenotypic screening of the 295 isolates from the strain collection revealed two cefoxitin screening–positive isolates (0.7%) with mean diameters of 14 mm (range 13–14 mm) and 15 mm (all 15 mm), respectively. Fourteen isolates (4.7%), including the two that were cefoxitin screening positive, showed growth on ChromID MRSA agar. Cefoxitin diameters of the other 12 isolates were in the susceptible category (range 27–32 mm); thus, sensitivity of the ChromID MRSA agar for phenotypic methicillin resistance detection was 100% and specificity was 95.9%.

Further analysis by molecular testing was performed on these fourteen isolates. The two cefoxitin-resistant isolates were positive for *mecC* and negative for *mecA* by conventional PCR as well as with the new Genspeed MRSA test. In accordance with their susceptibility to cefoxitin, the other 12 isolates carried neither *mecA* nor *mecC*. Additionally, the four strains that were received in our capacity as reference laboratory were analyzed. All four isolates had a positive cefoxitin screening test with diameters of 17, 18, 20 and 21 mm, respectively. Presence of *mecC* and absence of *mecA* was confirmed with both PCR assays, and *lukF-PV* was not detected in any of the strains.

Analysis of *spa* sequencing data showed four different *spa* types among the six isolates: t5930 (*n* = 2), t1535 (*n* = 2), t843 (*n* = 1) and t3256 (*n* = 1). Whole genome sequencing of all isolates confirmed that each isolate encoded *mecC* within an SCCmec type XI. Sequence types derived from the genome sequences included three ST130, a single-locus variant of ST130 and two ST599. All six isolates were positive for the genes encoding leukocidin ED (*lukED*) (GenBank accession no. Y13225) and exfoliative toxin A (*eta*) (GenBank accession no. CAQ49592). The four isolates belonging to CC130 additionally carried the epidermal cell differentiation inhibitor B gene (*edin-B*) (GenBank accession no. AHC54577) and the exfoliative toxin D gene (*etd2*) (GenBank accession no. HF563069). The two ST599 isolates were also positive for the toxic shock syndrome toxin-1 (*tst*) (GenBank accession no. Q9F0L4) and the enterotoxin-L (*sel*) gene (GenBank accession no. CAI80052) (Table 1). Other than *mecC* and *blaZ,* no other acquired resistance genes were identified.

Analysis of *Sma*I pulsed-field gel electrophoresis patterns showed the strains to be unrelated, with the exception of isolates 5590/2012 and 5625/2012, which were isolated in the same area of Austria within 8 weeks of each other. The Dice coefficient for this pair was 85.9%, indicating possible relatedness.

Subsequently, the six *mecC* MRSA were subjected to antimicrobial susceptibility testing and yielded susceptible results for all non-β-lactam antibiotics (Table 1). Two isolates (5127/2010 and 5752/2013) had oxacillin MICs below the breakpoint usually used to infer methicillin resistance. Vitek 2 identified all six isolates correctly as *S. aureus* and gave a positive result for the cefoxitin screen in five isolates, whereas all isolates were given a susceptible result for oxacillin. One isolate (5752/2013) was identified as methicillin-susceptible *Staphylococcus aureus* (MSSA) with a negative cefoxitin screen also upon repetition of the test. The remaining susceptibility data of the AST GP card matched the results of manual testing.

**FIG. 1:**
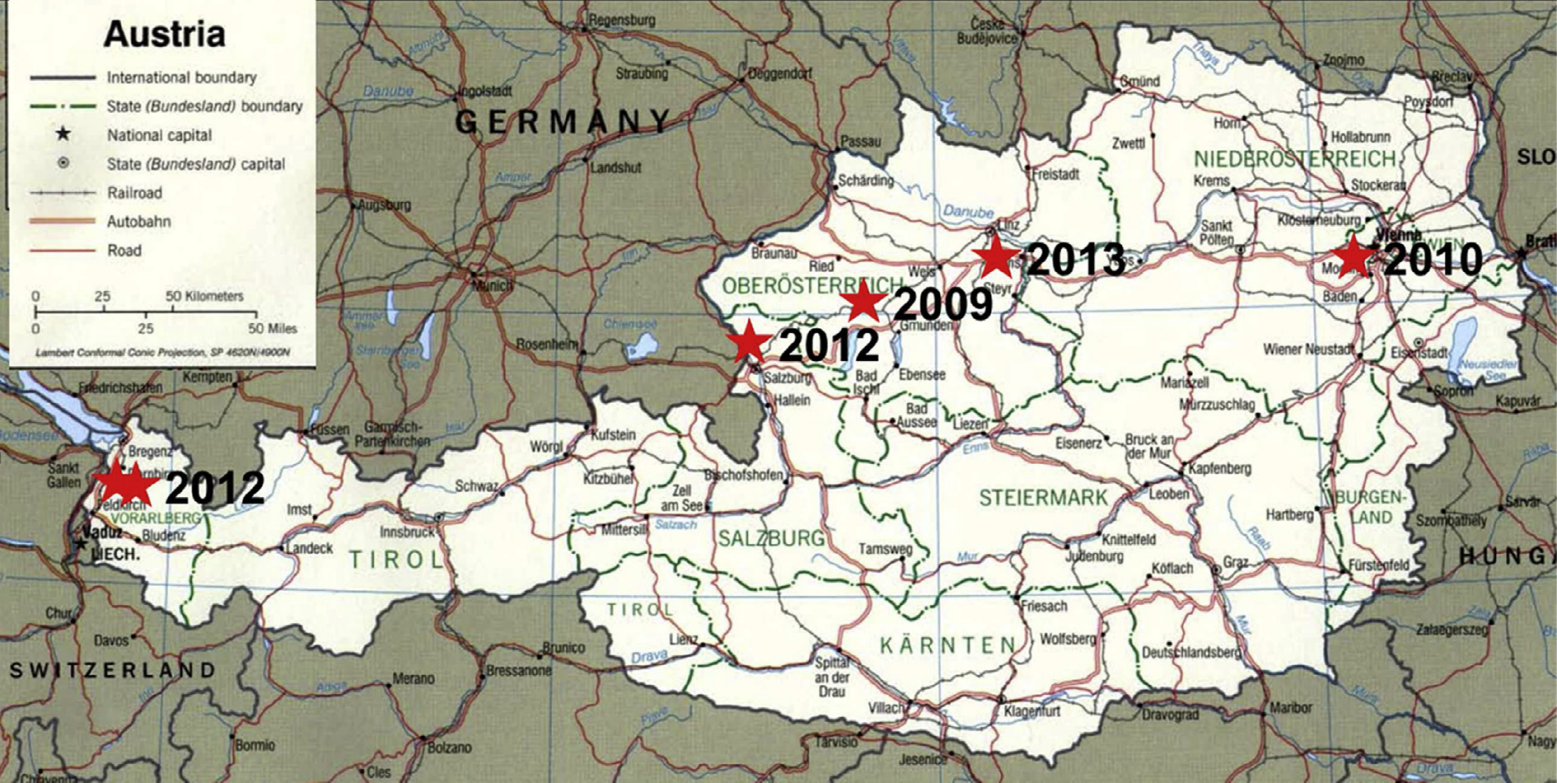
Location and year of isolation of six *mecC* methicillin-resistant *Staphylococcus aureus* isolates. Most patients lived in rural areas of Austria. (Map is a public domain file downloaded from http://www.mygeo.info/karten/austria_pol99.jpg.)

Clinical data are summarized in Table 2, and the geographic location of the laboratories that provided the *mecC* MRSA strains as well as the year of detection are indicated in Fig. 1.

## Discussion

To our knowledge, this is the first report concerning the detection of *mecC* MRSA in humans in Austria. We can confirm the presence of such strains back to the year 2009 resulting from the investigation of our strain collection. The only other data on *mecC* MRSA in Austria covers livestock- and wildlife-origin strains [20,21].

All *mecC*-positive isolates in this study were phenotypically recognizable as MRSA using cefoxitin in the Eucast disk-diffusion test, which has been shown to reliably detect these strains [22]. However, the built-in cefoxitin screen of the Vitek 2 GP AST card detected only five of the six isolates, which is in contrast to a recent study using the same system successfully on 62 *mecC* MRSA isolates [23]. However, the atypical Vitek 2 oxacillin-susceptible/cefoxitin-resistant profile of *mecC* MRSA as described by Cartwright *et al.* [23] was observed in the remaining five isolates. Isolate 5752/2013, which was misidentified as MSSA by Vitek 2, had the largest cefoxitin diameter (21 mm) of all isolates and thus was also challenging for disk-diffusion test reading. All isolates were universally susceptible to non-β-lactam antibiotics, which compares to data on *mecC* MRSA from other European countries [6,7,24].

Previous reports have shown reduced efficiency of growth on commercially available selective chromogenic media, also including ChromID agar [6], for ST130 *mecC* MRSA. In contrast, in our small sample also containing ST130 strains, all isolates grew well on ChromID agar. However, the cefoxitin MICs of our isolates were considerably higher than those reported by Cuny *et al.* [6] (all ≥16 mg/L). The published cefoxitin content of this medium is 4 mg/L [25,26], so efficient growth was not unexpected. Also, the observed false-positive rate of 4.1% is in line with published data [27].

Up to now, PCR methods for MRSA detection have successfully covered all known SCCmec types as a result of the high homology of the *mecA* gene. However, the newly discovered *mecC* element shares only 69% identity of its DNA sequence with *mecA*, leading to negative results using *mecA*-based PCR protocols and a need for additional primers to detect *mecC*. At the moment, not all commercially available assays cover this requirement. The Genspeed MRSA test that we used in this study can be completed in 75 minutes from culture; it can also be performed directly from swabs. It may be used as screening tool for colonization with *S. aureus* (MSSA) as well as *mecA* or *mecC* MRSA.

Four of the six *mecC* MRSA in our study belonged to CC130, which agrees with previously published data from other countries that also show CC130 to be predominant [1,2,7,17,24]. Analysis of whole genome sequences of six strains revealed that the CC130 isolates lacked pyrogenic toxin superantigen (PTSAg) encoding genes, such as the toxic shock toxin gene *tst* and staphylococcal enterotoxin genes. However they were all positive for exfoliative toxin genes (*eta* and *edinB*). The occurrence of *mecC* in MRSA isolates belonging to ST599 is relatively uncommon, but the two isolates in our study were positive for the PTSAg genes *tst* and *sel,* which matches the toxin gene profile of previously published ST599 *mecC* MRSA isolates [5,28]. Four different *spa* types were recovered in this study, all of which have previously been reported among *mecC* MRSA. t843 was the dominant *spa* type of *mecC* MRSA in a range of reports [4,7,17] and *spa* types t1535 and t3256 have also been described [7,24], whereas t5930 has so far only been mentioned in a single report from France [5].

The capability of *mecC* MRSA to cause clinical disease in a wide range of patients has been established by several reports. In a Spanish case series, most patients were elderly men who were colonized, but a patient with lethal sepsis was also described [7]. Petersen *et al.* [4] reported on 112 *mecC* MRSA isolates from Denmark, with equal sex distribution and a mean patient age of 51 years. Interestingly, the majority of the isolates were from infections, mostly skin and soft tissue infection, and not from colonization screening. In a recent report on the prevalence of human *mecC* MRSA in England, more than half of the isolates were identified from screening samples [24], whereas in a case series from Germany, most strains were isolated from wounds but also included was an isolate from nosocomial pneumonia [6]. A French case report described a patient with mediastinitis and sternal osteitis due to *mecC* MRSA [9]. In two of our six clinically diverse study patients, *mecC* MRSA were identified via routine screening, but in the remaining four patients, it was clearly associated with their clinical presentation.

Overall, *mecC* MRSA do not seem to be highly prevalent in Austria at the moment, which reflects the situation in most other European countries [10]. On the other hand, the frequency of *mecC* MRSA has increased significantly to a proportion of 2% of the total annual human MRSA cases in Denmark [4]. As *mecC* is carried on a mobile genetic element (SCCmec), it has the potential to spread into different lineages [29–31]. Thus, reliable detection and monitoring of *mecC* MRSA with appropriate phenotypic and molecular screening and confirmation methods is needed.

## Conflict of interest

None declared.

## Figures and Tables

**TABLE 1:** Phenotypic and molecular typing data of six *mecC* methicillin-resistant *Staphylococcus aureus* isolates

	Isolate 4402/2009	Isolate 5127/2010	Isolate 5590/2012	Isolate 5625/2012	Isolate 5676/2012	Isolate 5752/2013
Disk diffusion test	Diameter (mm) (category)	Diameter (mm) (category)	Diameter (mm) (category)	Diameter (mm) (category)	Diameter (mm) (category)	Diameter (mm) (category)
Cefoxitin	18 (mean, R)	16 (mean, R)	21 (mean, R)	18 (mean, R)	17 (mean, R)	20 (mean, R)
Gentamicin	20 (S)	22 (S)	20 (S)	22 (S)	24 (S)	22 (S)
Erythromycin	26 (S)	26 (S)	24 (S)	28 (S)	30 (S)	30 (S)
Clindamycin	25 (S)	26 (S)	22 (S)	26 (S)	30 (S)	30 (S)
Tetracycline	25 (S)	26 (S)	22 (S)	27 (S)	25 (S)	30 (S)
Fusidic acid	30 (S)	31 (S)	26 (S)	30 (S)	30 (S)	30 (S)
Trimethoprim/sulfa	32 (S)	31 (S)	26 (S)	34 (S)	30 (S)	30 (S)
Rifampicin	31 (S)	30 (S)	27 (S)	32 (S)	30 (S)	30 (S)

**Gradient MIC test**	**MIC (mg/L)**	**MIC (mg/L)**	**MIC (mg/L)**	**MIC (mg/L)**	**MIC (mg/L)**	**MIC (mg/L)**

Ceftaroline	1 (S)	1 (S)	1 (S)	1 (S)	0.5 (S)	0.5 (S)
Vancomycin	1 (S)	1 (S)	2 (S)	1 (S)	1 (S)	2 (S)
Teicoplanin	1 (S)	1 (S)	2 (S)	1 (S)	1 (S)	0.25 (S)
Tigecycline	0.25 (S)	0.25 (S)	0.25 (S)	0.25 (S)	0.125 (S)	0.25 (S)
Linezolid	0.5 (S)	0.5 (S)	1 (S)	0.5 (S)	0.5 (S)	2 (S)
Daptomycin	0.25 (S)	0.125 (S)	0.125 (S)	0.125 (S)	0.25 (S)	0.25 (S)
Fosfomycin	1 (S)	1 (S)	0.5 (S)	1 (S)	0.5 (S)	2 (S)
Oxacillin	4	4	2	8	4	2
Cefoxitin	16	32	16	16	32	16

**Typing**						

Multilocus sequence	599	130	SLV of 130	130	599	130
Type						
Clonal complex	599	130	130	130	599	130
*spa* Type	t5930	t3256	t1535	t1535	t5930	t843
Virulence factor gene						
*tst*	+	−	−	−	+	−
*lukED*	+	+	+	+	+	+
*eta*	+	+	+	+	+	+
*etd2*	−	+	+	+	−	+
*edin-B*	−	+	+	+	−	+
*sel*	+	−	−	−	+	−

SLV, single locus variant; MIC, minimum inhibitory concentration.

**TABLE 2:** Clinical information of six *mecC* methicillin-resistant *Staphylococcus aureus* isolates

Characteristic	Isolate 4402/2009	Isolate 5127/2010	Isolate 5590/2012	Isolate 5625/2012	Isolate 5676/2012	Isolate 5752/2013
Patient age/sex	89/female	54/female	7/female	72/female	83/male	70/male
Site of isolation	Blood culture	Multisite screen	Wound swab (outer ear)	Wound swab (leg ulcer)	Nose screen	Blood culture
Underlying disease	Myelodysplastic syndrome, sepsis	Diabetes, Eczema	Otitis externa	Ulcus cruris	Stroke	Peripheral arterial occlusion disease, infected leg ulcer
Therapy	Unknown	Octenidine/mupirocin	Topical ofloxacin	Topical silver-sulfa-diazine/povidone-iodine	Mupirocin	Clindamycin iv
Outcome	Recovered	Eradication	Recovered	Improvement	Eradication	Recovered
Risk factors	Unknown	Unknown	Pet rabbit (not screened)	Unknown	Unknown	Unknown
Geographic location	Upper Austria	Vienna	Vorarlberg	Vorarlberg	Salzburg	Upper Austria

No contact could be established between patients.
